# Combined Oral Fentanyl Citrate and Midazolam as Premedication for Bone Marrow Aspiration and Biopsy in Patients with Hematological Malignancies: A Randomized, Controlled and Patient-Blinded Clinical Trial

**DOI:** 10.3390/jcm9020395

**Published:** 2020-02-01

**Authors:** Claudio Cerchione, Giovanni Martinelli, Marco Picardi, Novella Pugliese, Davide Nappi, Aniello Casoria, Angela Gravetti, Delia Cangini, Maria Benedetta Giannini, Sonia Ronconi, Giorgia Simonetti, Andrea Ghelli Luserna Di Rorà, Ugo De Giorgi, Mattia Altini, Sara Bravaccini, Ilaria Santoriello, Cristiano Minucci, Fabrizio Pane, Vincenzo Martinelli

**Affiliations:** 1 Hematology Unit, Istituto Scientifico Romagnolo per lo Studio e la Cura dei Tumori (IRST) IRCCS, 47014 Meldola, Italy; giovanni.martinelli@Irst.emr.it (G.M.); delia.cangini@irst.emr.it (D.C.); maria.giannini@irst.emr.it (M.B.G.); sonia.ronconi@irst.emr.it (S.R.); 2 Hematology, Department of Clinical Medicine and Surgery, AOU Federico II, 80131 Naples, Italy; marco.picardi@unina.it (M.P.); novypugliese@yahoo.it (N.P.); daviden@hotmail.it (D.N.); aniello.casoria@gmail.com (A.C.); fabrizio.pane@unina.it (F.P.); vincenzo.martinelli@unina.it (V.M.); 3 Hematology Unit, Azienda Ospedaliera di Rilievo Nazionale (AORN) Antonio Cardarelli, 80131 Naples, Italy; angelagravetti@gmail.com; 4 Biosciences Laboratory, Istituto Scientifico Romagnolo per lo Studio e la Cura dei Tumori (IRST) IRCCS, 47014 Meldola, Italy; giorgia.simonetti@irst.emr.it (G.S.); andrea.ghellilusernadirora@irst.emr.it (A.G.L.D.R.); sara.bravaccini@irst.emr.it (S.B.); 5 Department of Medical Oncology, Istituto Scientifico Romagnolo per lo Studio e la Cura dei Tumori (IRST) IRCCS, 47014 Meldola, Italy; ugo.degiorgi@irst.emr.it; 6 Healthcare Administration, Istituto Scientifico Romagnolo per lo Studio e la Cura dei Tumori (IRST) IRCCS, 47014 Meldola, Italy; mattia.altini@irst.emr.it; 7 Department of Anesthesiology, Federico II University, 80131 Naples, Italy; ilaria.santoriello@unina.it (I.S.); cristiano.minucci@unina.it (C.M.)

**Keywords:** bone marrow aspiration and biopsy, pain, analgesia, anxiolysis, fentanyl citrate

## Abstract

Bone marrow aspiration and biopsy (BMAB) is a painful procedure, and the routinely used local infiltration anesthesia (LIA) with lidocaine is unable to provide pain relief during the most uncomfortable phases. The primary endpoint of the present randomized, patient-blinded trial was to evaluate the efficacy of an opioid and benzodiazepine combination plus LIA (sedoanalgesia) in patients undergoing BMAB for hematological malignancies. The secondary endpoint was the safety of the procedure in an outpatient setting. Ancillary assessments were anticipatory anxiety related to pain recall in the event of re-biopsy, and adequacy of bone tissue harvested. Patients were randomly assigned to one of 2 arms to receive either sedoanalgesic placebo plus LIA (standard group) or oral fentanyl citrate 200 μg plus oral midazolam 5 mg plus LIA (combo group) during BMAB. Pre-procedural anxiety and procedural pain were assessed according to the Numerical Rating Scale (NRS: 0–10), dividing the time of the procedure into five intervals (T0, T1, T2a, T2b and T3) and evaluating the degree of discomfort at each time (T) in both groups. One hundred and sixteen patients were eligible for the study. At T2b (time of biopsy) and T3 (time after biopsy), a significantly lower perception of pain was registered in the combo group. Moreover, there were no significant sedoanalgesia-related side-effects. Finally, histological specimens were higher in quality in the combo group. Sedoanalgesia was highly effective in reducing pain during biopsy, diminished anticipatory anxiety in patients undergoing re-biopsy and led to fewer non-diagnostic specimens being harvested.

## 1. Introduction

Bone marrow aspiration and biopsy (BMAB) is a medical procedure commonly used for the diagnosis and follow-up of several hematological and non-hematological diseases [1,2]. In adults, BMAB consists in bone marrow aspiration and bone trephine biopsy, usually performed on the posterior iliac crest to reduce patient discomfort. Data on BMAB-related morbidity and mortality show that this procedure is safe, with major adverse events a rare occurrence [3]. Thus, the pain associated with BMAB is the main issue for patients undergoing this procedure, making its relief an important objective for physicians. Analgesia for BMAB is commonly provided by local infiltration anesthesia (LIA) alone [2]. Given the difficulty in achieving sufficient anesthesia in bone and bone marrow, BMAB is often a painful, uncomfortable and anxiogenic experience for patients. Moreover, a prospective study by Degen et al. reported that patients who had previously experienced a painful BMAB often referred to it as ‘unbearable’ pain [4].

In the setting of hematological malignancies, BMAB provides comprehensive data on cytological and histological morphology, cytogenetics and molecular biology, all of which are useful for diagnosis, therapeutic decision-making and the formulation of prognosis. The 2008 World Health Organization (WHO) Classification of Tumors of Hematopoietic and Lymphoid Tissues recommends that 500 nucleated cells be counted on bone marrow smears in an area as close to the particle and as undiluted with blood as possible, and that bone trephine biopsy be taken at the right angle from the cortical bone and at least 1.5 cm in length to enable the evaluation of at least 5 partially preserved intertrabecular areas [2]. However, a state of pain, discomfort and anxiety experienced by the patient may render it impossible to respect the above criteria, resulting in the failure of tissue harvest. Premedication, combining an analgesic and anxiolytic (known as sedoanalgesia), administered both systemically and locally, may be effective in reducing the discomfort associated with BMAB, relieving anticipated anxiety from the pain experienced during a previous BMAB, and establishing optimal conditions that facilitate the harvesting of adequate specimens. In fact, there is still no clear evidence of an effective single-agent premedication capable of significantly reducing BMAB-associated pain and memory-related discomfort experienced by patients.

An average of 1000 BMABs are performed each year in the Hematology Unit of Federico II University, and around one-third of patients require repeat biopsies to monitor the underlying hematological disease. We performed a randomized, controlled, patient-blinded study with the main objective of assessing the effectiveness of an oral analgesic plus anxiolytic plus LIA compared to LIA alone in reducing discomfort in patients undergoing BMAB in an outpatient setting. A secondary objective was the safety of sedoanalgesia, especially in an outpatient setting, as was the case in our study. Useful ancillary data were also collected, i.e., which method of analgesia (standard or combo) was preferred by patients who had already undergone BMAB, and whether sedoanalgesia influenced the quality of the biological specimens harvested. A review of the literature was also carried out to compare our data with those of other authors describing similar combinations of analgesic/anxiolytic agents.

## 2. Materials and Methods

### 2.1. Trial Design and Participants

Eligible patients were randomly assigned at a 1:1 allocation ratio to receive BMAB using one of 2 analgesic methods, i.e., sedoanalgesic placebo plus LIA (standard group) or premedication (opioid + benzodiazepine agents) plus LIA (combo group).

Patients were required to meet the following eligibility criteria: age ≥18 years, confirmed diagnosis of hematological malignancy and indication to perform BMAB in an outpatient unit. Exclusion criteria were concomitant psychiatric conditions, major neuropathy, severe respiratory impairment, positive medical history for allergy to benzodiazepine or opioid agents and pregnancy and/or breast-feeding.

This was a single-center study. Eligible patients were registered at the Hematology Division Office of Federico II University of Naples, where the trial was designed and approved by the University Ethical Committee of in early 2008 (registration no. 101/08). The study was conducted in accordance with the ethical standards laid down in the 1964 Declaration of Helsinki. All patients signed the informed consent before enrolment.

### 2.2. Interventions

Patients randomized to the combo group received 200 μg oral transmucosal fentanyl citrate (Actiq^®^; Teva Pharmaceuticals Europe B.V., Haarlem, The Netherlands) plus 5 mg midazolam (oral Midazolam Accord Healthcare^®^, Accord Healthcare Limited, Middlesex, UK) before the BMAB. The benzodiazepine was given 30 min before the opioid, which was dissolved in the mouth over 10 min. The same opioid and a benzodiazepine placebo was administered to patients randomized to the standard group. After around 10 min, both groups underwent routine administration of LIA (10 mL of 2% lidocaine was injected subcutaneously through a 21-gauge needle into the periosteum at the iliac crest). The BMAB was performed on the left posterior superior iliac spine, with the patient in a standard prone position [2]. A single physician (V.M.) with long-standing experience in BMABs performed the procedure. The puncture area was disinfected and draped using the Trap system HS^®^ kit (HS Hospital Service, Latina, Italy) and an 11-gauge, 100 mm needle was used, regardless of the gender, age or physical build of patients. After the procedure, the puncture site was covered with a bandage and the patient rested in bed for 30 min, monitored by the hematologist who performed the biopsy. Main vital parameters such as blood pressure, heart rate, peripheral oxygen saturation were measured at each time point of the BMAB to check for the onset of adverse events. Cognitive function was measured before and 30 min after the procedure and any side-effects recorded. Patients were instructed not to drive or operate machinery and not to remain alone for 6 h after discharge. Finally, a telephonic interview was performed 24 h later to check for the onset of late side-effects.

### 2.3. Pre-Procedural Interview

A pre-procedural interview was carried out one week before the BMAB and the following information was collected from all patients: (1) general clinical status at that moment and during the previous weeks; (2) use of drugs in the previous days associated with an increased risk of bleeding (e.g., Nonsteroidal anti-inflammatory drugs (NSAIDs), anticoagulants, etc.); and (3) past clinical history of allergy to anesthetics or other drugs. In the event of chronic therapy with aspirin or anticoagulants, the patient was advised to switch temporarily to heparin-based treatment. Patients with a clinical history of allergies were prescribed a 3-day prophylactic course of corticosteroids and anti-histaminic drugs.

### 2.4. Primary and Secondary Outcomes

The study aimed to demonstrate the superiority of the combined opioid/benzodiazepine therapy plus LIA over LIA alone in reducing patient discomfort during the most painful phases of BMAB. The degree of analgesia to use in each group was defined using the Numeral Rating Scale (NRS: 0–10; 0 = no anxiety or pain, 10 = worst possible anxiety or pain) [5] by dividing the procedure into 5 main time (T) intervals (T0, baseline; T1, start of procedure and administration of sedoanalgesia; T2a, bone marrow aspiration; T2b, trephine biopsy and T3, five minutes after the end of the procedure) and evaluating the degree of discomfort at each time. A research nurse also asked patients at the beginning and end of the procedure to grade anxiety and pain according to the NRS (location of pain was also recorded). At T3, all patients were asked to complete a questionnaire on efficacy, satisfaction and comfort rated according to a 4-point scale.

The secondary endpoint of this study was the safety of sedoanalgesia used in an outpatient setting. Ancillary data were also recorded, i.e., a post hoc analysis in randomized in combo patients previously submitted to BMAB with LIA alone to evaluate if they preferred the standard or combo method of anesthesia, and whether sedoanalgesia influenced the quality of the biological specimens harvested. Analysis of the adequacy of BMAB samples harvested was performed by 2 expert pathologists with more ten years’ experience in the field and based on the criteria of the 2008 WHO Classification of Tumors of Hematopoietic and Lymphoid Tissues [6].

### 2.5. Sample Size

We tested the hypothesis that the addition of an oral regimen combining an analgesic with an anxiolytic (opioid and benzodiazepine, respectively) plus LIA could reduce the pain score of patients undergoing BMAB with respect to LIA alone. Based on previous studies, we estimated a reported pain level of 0–2 in 78% of patients in the standard group [7]. To detect a rate > 10% patient improvement (for the superiority test) in the combo group, 93 patients were needed when using a two-sided type I error of 5% and 99% statistical power. Assuming a dropout rate of 10%, we set a final sample size of at least 47 ± 5 patients in each group.

### 2.6. Randomization and Blinding

After the participants had signed the informed consent, a random allocation sequence was generated by the study statistician using a computerized system (procedure outlined elsewhere).

### 2.7. Statistical Analysis

The χ^2^ test was performed to compare proportions for clinical and histological characteristics, and complication rate, while the *t* test was used to compare the quantitative variables of clinical characteristics between the two groups (*p*-values below 0.05 were statistically different).

## 3. Results

### 3.1. Participants and Recruitment

**Figure 1:**
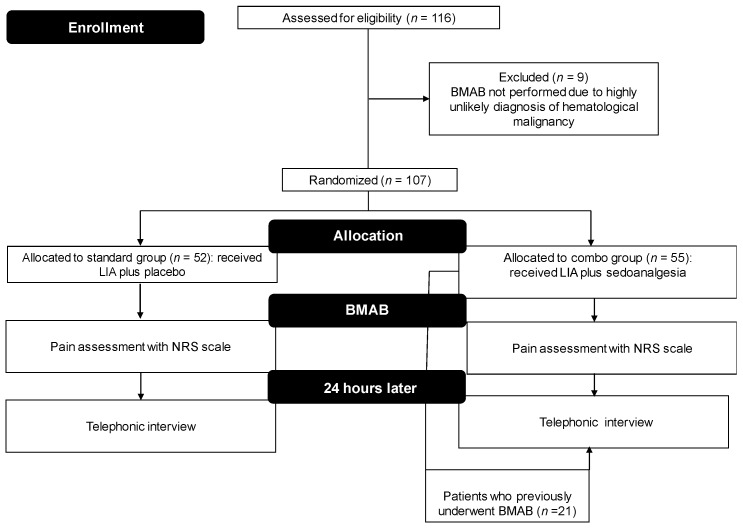
Flow chart of the trial. BMAB, bone marrow aspiration and biopsy; LA, local anesthesia.

From July 2008 to July 2015, 116 patients were enrolled in the trial, of whom 9 (5.2%) were excluded because the diagnosis of hematological malignancy was highly unlikely after laboratory and instrumental assessments. Fifty-two (48.6%) patients received LIA and placebo administered orally (standard group), while 55 (51.4%) underwent LIA plus 5 mg midazolam (oral administration) and 200 µg transmucosal fentanyl 30 min before the procedure (combo group). None of the patients were lost to follow-up or withdrew consent to participate in the study. Thus, 107 patients were included in the final analysis. Baseline characteristics of randomized patients are shown in Table 1 and a brief flow-chart of the trial design is provided as Figure 1. Eighteen (34.6%) patients in the standard group and twenty-one (38.1%) in the combo group had previously undergone BMAB.

### 3.2. Efficacy

#### 3.2.1. Questionnaires and Interview Results—Primary Endpoint

**Figure 2:**
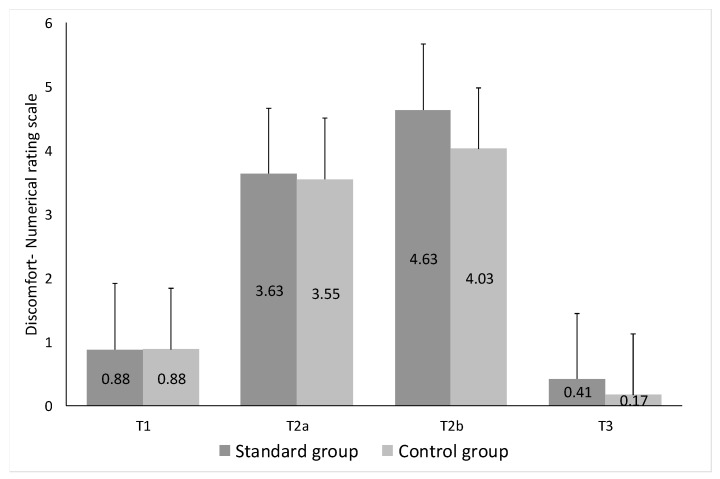
Pre-procedural anxiety and procedural pain were assessed according to the Numbered Rating Scale (NRS: 0–10), dividing the time of the procedure into five intervals (T0, T1, T2a, T2b and T3) and evaluating the degree of discomfort at each time (T) in both groups.

The following information was obtained from the completed questionnaires and post-procedure telephonic interviews: the average level of pain at T1, measured by Numerical Rating Scale (NRS), was 0.87 for the standard group and 0.88 for the combo group; at T2a, 3.63 and 3.54, respectively; at T2b, 4.63 and 4.0, respectively (*p* < 0.05); and at T3, 0.41 and 0.16, respectively (*p* < 0.05). As expected, the most painful phases corresponded to T2a and T2b, i.e., bone marrow aspiration and trephine biopsy, respectively. There were no significant differences between the 2 groups in relation to start of procedure + aspiration (T1 + T2a), biopsy (T2b) and 5 min after the procedure (T3) (Figure 2).

#### 3.2.2. Effect on Cognition and Side-Effects—Secondary Endpoint

Patients in the combo group experienced mild conscious sedation with a brief period (15–30 min) of deep relaxation that corresponded to the duration of the procedure. None of the group recalled the event clearly or reported experiencing pain linked to the BMAB. Only 2 patients showed typical opioid-related side-effects (nausea or nausea/vomiting–grade 1/2) that lasted < 4 h in either case and only one required the administration of an antiemetic drug.

#### 3.2.3. Ancillary Endpoints

When interviewed telephonically after 24 h, all the 21 patients in the combo group who had previously undergone a BMAB (less than one year before) without administration of an oral analgesic and anxiolytic did not describe the previous day’s procedure as an unpleasant experience and stated that they preferred the new drug combination.

#### 3.2.4. Adequacy of BMAB—Harvested Specimens

Taking into account the literature data on the topic, the length and quality of the bone marrow specimens in the combo group were superior (medium measured length 21.2 mm [range 16.4–23.9 mm]) to those of the standard group (median measured length 17.9 mm [range 10.6–21.8 mm]). The longer specimens harvested in the combo group also resulted in a higher quality of the diagnostic process: all 55 combo patients (100%) had a fully conclusive pathological diagnosis compared to 48 (92.3%) in the standard group. Primary and secondary endpoint results are summarized in Table 2.

A cost-effectiveness analysis has also been performed (Table 3): This confirms how affordable is the sedoanalgesia procedure in whatever Hematology Unit.

## 4. Discussion

There is no clear evidence indicating a single effective method of premedication before BMAB in adults, and no formal guidelines for using analgesia/anxiolysis in this setting. Our preliminary results confirmed the different subjective perception of pain in the 2 patient groups, one treated with the LIA plus sedoanalgesia and the other with LIA alone, also underlining a high level of satisfaction and general comfort in patients in the combo group. The primary endpoint of this superiority study was thus reached: the degree of pain reduction in sedoanalgesia patients compared to those receiving standard premedication differed significantly. Given that a less painful experience associated with BMAB can notably improve the quality of life in patients with a chronic disease, sedoanalgesia can be considered superior to LIA alone as a pre-procedural anesthetic method. Compared to others papers, the presence of a single operator performing the procedure in our study overcame the problem of different pain perception deriving from different experience levels of the physician performing the BMAB. Hence, the result of a different degree of discomfort can only be ascribed to the efficacy of the medications. In addition, having the same operator perform the procedure each time overcame potential operator-dependent bias.

The clinical importance of our data is evident because all of the patients (*n* = 21) in the combo group (who had previously undergone BMAB with LIA alone), when given the choice of sedoanalgesia or standard LIA, chose to repeat the combination premedication.

In addition to showing the efficacy of fentanyl citrate, our results also underlined its versatility and safety as an analgesic component of the premedication used in our trial. Widely employed for episodes of breakthrough cancer pain and for noncancer pain-related events, its pharmacological features also make it suitable for a short-term sedation. The opioid is administered as a sweetened lozenge with an integral oromucosal applicator that allows part of the drug to avoid first-pass metabolism and become rapidly available through the oral mucosa [8]. Several studies on the safety of transmucosal fentanyl have focused mainly on its use for the treatment of cancer-related pain or as premedication in children undergoing invasive procedures, the most frequently reported side-effects being typical opioid-related such as nausea, vomiting, dizziness and confusion [9,10,11,12]. Only 2% of our patients showed typical adverse opioid-related events (nausea and vomiting–grade 1/2) after the procedure. Its rapid onset of action, the short duration of its effect, and the lack of major side-effects make fentanyl citrate ideal for premedication in minimally invasive procedures such as BMAB.

Another advantage of sedoanalgesia before BMAB was the better quality of bone specimens obtained with premedication compared to LIA alone. The median length of the specimens obtained was 17.9 mm in the standard group (range 10.6–21.8 mm) and 21.2 mm in the combo-group (range 16.4–23.9 mm), which is above the optimal threshold of 20 mm [2]. This obviously had important consequences such as a reduction in non-diagnostic specimens (thus avoiding the need for repeat BMAB) and, primarily, improved quality of the histological diagnosis.

Some authors have used various methods and combinations of analgesia and anxiolysis in an attempt to reduce the pain from BMAB, obtaining different results. Several combinations of sedoanalgesia have been used with different administration modalities, together with different pain measuring scales, timing to monitor parameters and number of operators. In one of the first large studies focusing on the contribution of a single anxiolytic agent (oral lorazepam 4 mg) added to LIA, pre-medication significantly reduced the perception of pain after 24 h and the memory of the uncomfortable event [13]. A combination of oral lorazepam plus oral hydromorphone (variable dosages) together with local anesthesia was assessed by Thomas et al. who reported a general reduction in the pain experienced by patients [14]. Some authors also compared Entonox™, a volatile sedative and analgesic agent (50:50 mix of nitrous oxide and oxygen) with LIA alone or with intravenous midazolam, Entonox proving more effective than the benzodiazepine or LIA [15,16]. Another study on the prevalence and prevention of pain during BMAB evaluated the action of tramadol vs. placebo in the interventional phase, reported a reduction in the number of patients experiencing at least moderate pain during aspiration [17]. Talamo et al. compared LIA alone (lidocaine 50 mg) with LIA plus oral oxycodone 10 mg + oral lorazepam 2 mg + oral acetaminophen 650 mg, obtaining a significant reduction in the perception of pain in the combo group compared to the standard group, but not achieving a major clinically relevant effect (average pain level was similar) [7].

## 5. Conclusions

Pain is common in cancer patients and physicians should not neglect this component of the disease because of its potentially dramatic effect on quality of life. Although there are some known non-modifiable factors associated with higher pain levels, such as younger age, body mass index > 30 kg/m^2^ and female gender [18,19], there are no specific interventions for their management. Experience in performing a BMAB is associated with a lower level of pain because a skilled physician can notably reduce the length of the procedure and minimize the need for a repeat biopsy [19]. Anticipatory fear of a painful procedure such as BMAB can substantially increase the perception of pain and play a key role in the discomfort felt by the patient, creating a general state of anxiety that can negatively affect quality of life. Patients receiving premedication with benzodiazepines tend to be more willing to undergo other BMABs than those undergoing LIA alone [20], suggesting that retrograde amnesia induced by the sedative component of the premedication probably plays a major role in reducing pain and improving comfort of the procedure as the analgesia component. Our results show that premedication with oral midazolam plus transmucosal fentanyl citrate can effectively reduce both pain and pain-recall anxiety without major adverse events in an outpatient setting. Furthermore, allowing patients to choose whether to add sedoanalgesia to standard LIA helps them to go through a BMAB with greater serenity and minor discomfort, both of which are important for the diagnosis and management of hematological malignancies. Cost-effectiveness analysis showed that this procedure of great advantage for patient quality of life, is reasonably affordable for the majority of hospitals in Europe. In conclusion, physicians must not deny patients the opportunity for sedation as it is impossible to estimate the degree of pain produced by a procedure involving non sedated tissue such as bone or bone marrow.

## Figures and Tables

**Table 1:** Baseline patient characteristics.

Patient Characteristics	Standard Group	Combo Group
No. of patients	52	55
Male/Female	25/27	26/29
Median age, years (range)	61 (19–84)	60 (21–82)
**Type of hematologic malignancy**		
Non-Hodgkin’s lymphoma	24	20
Essential thrombocythemia	8	11
Polycythemia vera	8	10
Hodgkin’s lymphoma	6	7
Primary myelofibrosis	4	4
Chronic myeloid leukemia	2	3

**Table 2:** Primary and secondary endpoints. ^1^ Number of patients (percentage).

Endpoint	Standard Group	Combo Group
**NRS Pain Assessment**		
*T1*	0.87	0.88
*T2a*	3.63	3.54
*T2b*	4.63	4.0
*T3*	0.41	0.16
**Painful experience recalling ^1^**	52 (100%)	0
**Opioid side effects ^1^**	0	2 (3.63%)
**Specimen medium lenght (mm)**	17.9	21.2
**Conclusive histologic diagnosis ^1^**	48 (92.3%)	55 (100%)

**Table 3:** Cost-effectiveness analysis (All digits are Euro).

	Actiq^®^	Midazolam	Biopsy Needle	Total Charge	Total Charge Per Patient	Total Net Charge Difference	Net Charge Difference Per Patient
Standard group	-	-	1568	1568	30.15	-	-
Combo group	266	56.375	1540	1862.375	33.85	**+294.375**	**+3.7**
